# Predictor characteristics necessary for building a clinically useful risk prediction model: a simulation study

**DOI:** 10.1186/s12874-016-0223-2

**Published:** 2016-09-21

**Authors:** Laura Schummers, Katherine P. Himes, Lisa M. Bodnar, Jennifer A. Hutcheon

**Affiliations:** 1 Department of Epidemiology, Harvard T.H. Chan School of Public Health, 677 Huntington Avenue, Boston, MA 02115 USA; 2 Department of Epidemiology, Graduate School of Public Health, and Department of Obstetrics, Gynecology, and Reproductive Sciences, University of Pittsburgh, A742 Crabtree Hall, 130 DeSoto Street, Pittsburgh, PA 15261 USA; 3 Department of Obstetrics, Gynecology, and Reproductive Sciences, Magee-Womens Research Institute, University of Pittsburgh, 300 Halket Street, Pittsburgh, PA 15213 USA; 4 Department of Obstetrics & Gynaecology, University of British Columbia, 4500 Oak Street C408, Vancouver, British Columbia V6H3N1 Canada

**Keywords:** Epidemiologic methods, Risk prediction model, Discrimination, Risk classification, Model performance, Area under the receiver operating characteristic curve

## Abstract

**Background:**

Compelled by the intuitive appeal of predicting each individual patient’s risk of an outcome, there is a growing interest in risk prediction models. While the statistical methods used to build prediction models are increasingly well understood, the literature offers little insight to researchers seeking to gauge a priori whether a prediction model is likely to perform well for their particular research question. The objective of this study was to inform the development of new risk prediction models by evaluating model performance under a wide range of predictor characteristics.

**Methods:**

Data from all births to overweight or obese women in British Columbia, Canada from 2004 to 2012 (*n* = 75,225) were used to build a risk prediction model for preeclampsia. The data were then augmented with simulated predictors of the outcome with pre-set prevalence values and univariable odds ratios. We built 120 risk prediction models that included known demographic and clinical predictors, and one, three, or five of the simulated variables. Finally, we evaluated standard model performance criteria (discrimination, risk stratification capacity, calibration, and Nagelkerke’s r^2^) for each model.

**Results:**

Findings from our models built with simulated predictors demonstrated the predictor characteristics required for a risk prediction model to adequately discriminate cases from non-cases and to adequately classify patients into clinically distinct risk groups. Several predictor characteristics can yield well performing risk prediction models; however, these characteristics are not typical of predictor-outcome relationships in many population-based or clinical data sets. Novel predictors must be both strongly associated with the outcome and prevalent in the population to be useful for clinical prediction modeling (e.g., one predictor with prevalence ≥20 % and odds ratio ≥8, or 3 predictors with prevalence ≥10 % and odds ratios ≥4). Area under the receiver operating characteristic curve values of >0.8 were necessary to achieve reasonable risk stratification capacity.

**Conclusions:**

Our findings provide a guide for researchers to estimate the expected performance of a prediction model before a model has been built based on the characteristics of available predictors.

**Electronic supplementary material:**

The online version of this article (doi:10.1186/s12874-016-0223-2) contains supplementary material, which is available to authorized users.

## Background

Given the intuitive appeal of individual-level risk prediction, there is growing interest in developing clinical risk prediction models. By tailoring each individual’s estimated risk of an adverse outcome according to their demographic and clinical characteristics, risk prediction models can distinguish high and low risk patients. This has the potential to improve health outcomes and reduce health care costs by identifying patients who would benefit from additional diagnostic procedures or treatment options, and those who would not.

While the statistical steps used to build prediction models are well described and increasingly well implemented, the literature offers little insight to researchers seeking to gauge whether a prediction model is likely to perform well for their particular research question. Pepe and colleagues [1] have demonstrated previously that a single predictor must have an extremely strong association with the outcome in order to sufficiently improve a model’s ability to discriminate cases from non-cases. However, it is difficult to generalize these findings to studies that aim to collect multiple predictors, ranging in prevalence and strength of association with the outcome. Thus, few researchers know how to assess the likelihood that their prediction model will perform adequately a priori based on the characteristics of the predictors they expect to collect in their study, or the extent to which the addition of novel predictors will improve the performance of existing models.

Predicting an individual woman’s risk of developing preeclampsia in pregnancy, a leading cause of maternal and perinatal morbidity [2], is of particular interest in perinatal epidemiology. Women identified as high risk in early pregnancy may benefit from increased prenatal surveillance, referral to tertiary care centers with high risk specialists in maternal-fetal medicine, or treatment with antiplatelet agents such as aspirin [2, 3]. Ruling out a high risk of preeclampsia would avoid unnecessary surveillance and maternal anxiety [2]. Accordingly, several research groups have built clinical risk prediction models for preeclampsia, each using commonly available demographic and clinical characteristics coupled with novel predictors unique to their data (e.g., biomarkers or imaging studies) [4–9]. Despite considerable clinical detail in these data sets, none of the models demonstrated sufficient performance for routine use in clinical practice.

Using the example of preeclampsia, we evaluated performance criteria (discrimination, risk stratification capacity, and calibration) of a model built using standard demographic and clinical predictors. We then augmented this data with simulated predictors ranging in prevalence and strength of association with preeclampsia, and built multiple clinical prediction models that incorporated one, three, or five of these new variables. The objective of this study was to guide the development of new risk prediction models by establishing the performance of risk prediction models under a wide range of predictor prevalence values and univariable odds ratios with the outcome of interest.

## Methods

Our study population included all overweight or obese women (body mass index ≥25 kg/m^2^) who gave birth to infants weighing at least 500 g or of at least 20 completed weeks of gestation in British Columbia, Canada from April 1, 2004 to March 31, 2012. We restricted to overweight and obese women because they are most likely to receive pre-pregnancy counselling on modifiable risk factors for adverse pregnancy outcomes such as preeclampsia, and are thus a population for whom a risk prediction model might be most useful. Data were obtained from the British Columbia Perinatal Data Registry [10], a high quality population-based data source administered by Perinatal Services BC that contains abstracted linked maternal and newborn antenatal and delivery admission medical record data [11]. Preeclampsia was identified using the International Classification of Diseases Version 10 (ICD-10) codes O11, O13-O16.

We built a logistic regression model predicting risk of pre-eclampsia using the following demographic and clinical characteristics: prepregnancy body mass index, maternal height, maternal age, parity, and smoking status. This is our “original model”. Assumptions of linearity were assessed for continuous variables (prepregnancy BMI, height, and maternal age). Linear, quadratic, categorical, and restricted cubic spline transformations were considered, and the transformation that minimized the Akaike Information Criterion (AIC) was selected. We used a ‘full model’ variable selection approach, in which all variables expected to predict preeclampsia on a priori grounds were included in the logistic regression model. This method is known to minimize bias that can be introduced by selecting variables according to statistical criteria [12]. Multi-collinearity between predictors was examined using Variance Inflation Factors, with a value >3 as an indicator of multicollinearity.

To determine the predictor characteristics necessary for a clinical prediction model to perform well in terms of discrimination and risk stratification capacity, we then augmented this “original model” with additional simulated predictors of preeclampsia, as might occur with attempts to improve current preeclampsia prediction models by adding biomarkers such as PlGF [13] or other novel predictors, such as sonographic imaging of placental morphology [14] that could potentially improve the performance of existing models. The prevalence values of the simulated predictors were set to 5 %, 10 %, 20 %, or 40 %, and strengths of association (univariable odds ratios) were set to range from 1 to 16. All simulated predictors were binary and independent of one another. We built clinical prediction models that included our original predictors, as well as one, three, or five of the simulated predictors. We then built 120 models to achieve every possible combination of these prevalence and odds ratio values. We repeated variable generation and all model building steps for the models built with the new variables 5000 times to account for variability in the random draws from the normal distribution used to create each simulated variable.

### Evaluation of model performance

We evaluated calibration of our models visually by comparing observed versus expected risks and formally using the Hosmer-Lemeshow goodness-of-fit test [12]. Discrimination was assessed using the c statistic (the area under the receiver operating characteristic curve, AUC, for binary outcomes), where 0.7 is commonly used to indicate minimally acceptable discrimination [12, 15]. We extended our assessment of discrimination by examining the proportion of the population classified into a risk stratum in which the likelihood ratio was greater than 10 or less than 0.1 [16]. Likelihood ratios were calculated by dividing the percentage of women with preeclampsia in each risk group by the percentage of women without preeclampsia in that risk group. The proportion of variability in the outcome that was explained by the predictors was measured using Nagelkerke’s r^2^, a summary indicator of model performance [17].

We also examined risk stratification capacity, which reflects the extent to which the model is able to divide patients into groups with clinically distinct risk profiles (i.e., high risk vs. low risk) [18]. These risk profiles are intended to alter women’s clinical management. Risk stratification capacity is most often assessed using deciles of predicted risk, which reflects arbitrary cutoffs based on statistical characteristics of the study population. Instead, we opted to use groupings of predicted risk that would reflect thresholds for treatment or surveillance decisions in clinical practice. We measured risk stratification capacity by assessing the proportion of the population classified into a clinically distinct risk group, defined as predicted risk greater than 15.0 % or less than 3.0 %. Given a population average risk of 8.4 %, these thresholds were determined by a maternal-fetal medicine physician (KPH) as the thresholds above or below which clinical management would be altered by the prediction score (that is, women with a predicted risk of >15.0 % would likely be managed as ‘high risk’, women with a predicted risk <3.0 % would likely be managed as ‘low risk’, while predicted probabilities of 3.0–15.0 % would be considered uninformative because they are clinically equivalent to the risk estimated in the absence of a model (8.4 %)).

Model overfitting, or optimism, was evaluated with 200 bootstrap samples drawn with replacement from the original sample. We repeated all model building steps to fit the model in each bootstrap sample. Average optimism (the average of the difference between the observed AUC in the study population and the AUC in each bootstrap sample) was subtracted from the AUC in the study population to calculate the optimism-corrected AUC [12]. We followed these steps for the original model, as well as for all models including simulated predictors.

### Sensitivity analysis

We conducted sensitivity analyses using different risk group definitions (2.0 %, 2.5 %, 18.0 %, 20.0 %) to evaluate how sensitive our findings were to this definition. To ensure that the performance of this prediction model did not reflect unique characteristics of preeclampsia, we built clinical prediction models for several other adverse pregnancy outcomes as sensitivity analyses. These outcomes were gestational diabetes, spontaneous preterm delivery before 32 weeks, indicated preterm delivery before 37 weeks, macrosomia, shoulder dystocia, cesarean delivery, postpartum hemorrhage requiring intervention to control bleeding, maternal mortality/severe morbidity, stillbirth, NICU stay ≥48 h, and in-hospital newborn mortality (see Schummers [19] for detailed outcome definitions). In addition to those predictors included in the model for preeclampsia, we included additional outcome-specific predictors in some models (see footnote of Additional file 1 for complete list). All analyses were conducted using Stata Version 12.0 [20].

## Results

Of the 334,861 births in British Columbia during the study period, 229,387 had available data on prepregnancy body mass index. Of these, the 75,225 overweight or obese women (body mass index ≥25) were included in this analysis. Table 1 presents the prevalence and odds ratios of the predictors of preeclampsia observed in our data. Predictors in our data ranged in prevalence from 0.4 % (history of neonatal death) to 43.3 % (nulliparity). Crude odds ratios ranged from 0.8 (history of stillbirth or spontaneous abortion) to 2.9 (pre-existing diabetes).

**Table 1:** Clinical characteristics and risk factors for preeclampsia included in clinical prediction model in our data set and those from a previously published cohort study of preeclampsia risk

Predictors in our data set		
	Prevalence n (%)	Crude odds ratio (95 % CI)
Maternal age ^a^		
<20	1,624 (2.2)	1.0 (0.8, 1.2)
20–29	32,140 (42.7)	REF
30–40	38,444 (51.1)	1.0 (0.9, 1.0)
≥40	3,017 (4.0)	1.4 (1.3, 1.6)
Prepregnancy body mass index ^a^		
25–29	46,979 (62.5)	REF
30–34	17,692 (23.5)	1.6 (1.5, 1.7)
35–39	6,968 (9.3)	2.1 (1.9, 2.3)
≥40	3,586 (4.8)	1.8 (2.5, 3.1)
Maternal height <60 in.	4,280 (5.7)	0.9 (0.8, 1.0)
Nulliparity	32,571 (43.3)	2.5 (2.4, 2.6)
Pre-existing diabetes	769 (1.0)	2.9 (2.4, 3.5)
Smoking	8,411 (11.2)	0.9 (0.8, 1.0)
History of stillbirth	713 (0.9)	0.8 (0.6, 1.1)
History neonatal death	281 (0.4)	1.0 (0.6, 1.5)
History of spontaneous abortion	18,046 (24.0)	1.0 (0.9, 1.0)

^a^Prepregnancy body mass index and maternal age at birth were modeled using restricted cubic splines

The Hosmer-Lemeshow goodness-of-fit test indicated adequate goodness of fit (calibration), with *p* = 0.33. Likewise, visual examination of observed versus predicted risks according to the original model indicated adequate calibration (data not shown). As shown in Table 2, the original model had poor risk stratification capacity, with only 19.2 % of the population classified into clinically distinct high or low risk groups (11.5 and 7.7 %, respectively). None of the strata had informative likelihood ratios (i.e., all likelihood ratios were between 0.1 and 10). Our original risk prediction model had an AUC of 0.68, slightly below the 0.7 value widely used as a threshold to indicate adequate discrimination performance [12]. The optimism-corrected AUC was also 0.68 (see Additional file 1), indicating minimal overfitting. Similarly, overall model performance appeared poor, with a Nagelkerke’s r2 indicating that this model explained only 7.2 % of the variability in preeclampsia risk.

**Table 2:** Risk stratification capacity of the original model: observed vs. predicted risk

Predicted risk (%)	No. of births per stratum (% of sample)	Observed risk (%)	Likelihood ratio (95 % CI)
<3.0	5,788 (7.7)	134 (2.3)	0.3 (0.2–0.3)
3.0–5.5	21,654 (28.8)	876 (4.0)	0.4 (0.4–0.4)
5.5–12.0 ^a^	33,178 (44.11)	2,846 (8.6)	1.0 (1.0–1.1)
12.0–15.0	5,960 (7.9)	800 (13.4)	1.7 (1.6–1.8)
>15.0	8,645 (11.5)	1,660 (19.2)	2.7 (2.6–2.9)
Total	75,225 (100.0 %)	6,313 (8.4)	-

^a^Given a baseline risk of 8.4 %, this category is clinically equivalent to the population average risk

The AUC obtained from the preeclampsia risk prediction model was comparable to those we built for other adverse pregnancy outcomes with AUCs ranging from 0.59 for stillbirth to 0.66 for gestational diabetes. All models exhibited minimal overfitting, with estimated optimism ranging from 0.02 to <0.01. See Additional file 1 for the observed and optimism-corrected AUCs for the prediction models for all outcomes we examined.

**Fig. 1:**
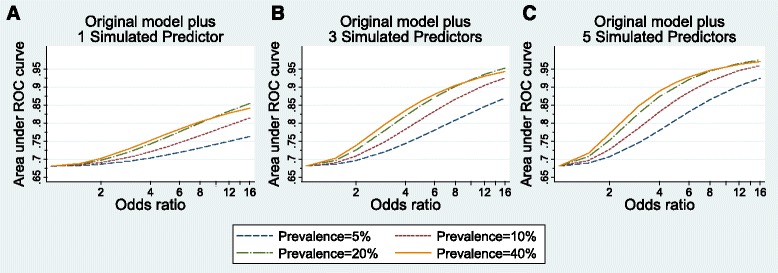
Discrimination performance (measured by area under Receiver Operator Characteristic curve) of risk prediction models according to simulated predictor characteristics. The original risk prediction model was augmented with simulated predictors with prevalence from 5 to 40 % and odds ratios ranging from 1 to 16: **a** one added simulated predictor per model; **b** three added simulated predictors per model; **c** five added simulated predictors per model

**Fig. 2:**
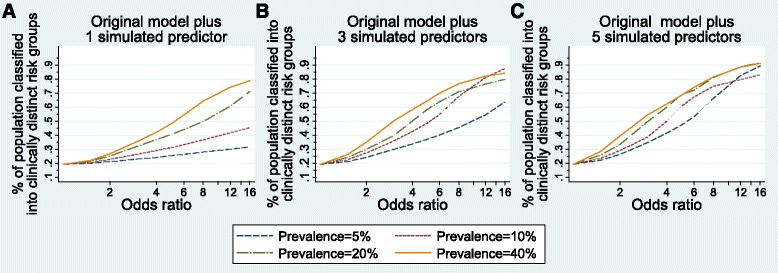
Proportion of population classified into a clinically distinct risk group (predicted risk <3.0 % or >15.0 %) from risk prediction models according to simulated predictor characteristics. The original risk prediction model was augmented with simulated predictors with prevalence from 5 to 40 % and odds ratios ranging from 1 to 16: **a** one added simulated predictor per model; **b** three added simulated predictors per model; **c** five added simulated predictors per model

The models built after adding simulated predictors demonstrate the prevalence and univariable odds ratio values necessary for a model to perform well in terms of discrimination, risk stratification capacity, and variability in outcome risk explained by the predictors. Figures 1, 2, and 4 show the model performance of each model by plotting the performance metric (AUC, proportion of the population classified into a clinically distinct risk group, and Nagelkerke’s r^2^, respectively) on the y-axes against the odds ratios of simulated predictors on the x-axes. Each curve in the figure represents a specific prevalence of the simulated predictors in the population, ranging from 5.0–40.0 %. Each sub-figure A-C represents the number of simulated predictors added to the models (one, three, and five, respectively).

The starting point for the AUCs of all models built with simulated predictors is 0.68, the observed AUC for the original model. From Fig. 1a (left), we see that the odds ratio for a single added predictor must be at least 6, and the prevalence at least 20.0 %, to achieve an AUC of 0.8. One common predictor (prevalence ≥20.0 %) with an odds ratio of 16 yields an AUC approaching 0.85. In Fig. 1b (center), we see that odds ratios for three common predictors (each with prevalence ≥20.0 %) need only reach a magnitude of 4 to produce a model with AUC of 0.8. Three common predictors with odds ratios of 16 can yield an almost perfect AUC, near 0.95. With five simulated predictors (Fig. 1c, right), rare predictors (5.0–10.0 % prevalence) can yield an AUC of 0.9, provided each odds ratio exceeds 10. Five common predictors (prevalence ≥20.0 %) with odds ratios of 3 to 4 can produce an AUC of 0.85, increasing to 0.95 as odds ratios increase. As with the original model, models including simulated predictors exhibited minimal overfitting, with AUC estimates remaining unchanged to 2 decimal places after correcting for optimism.

Figure 2 depicts the risk stratification capacity of each model after adding simulated predictors according to the proportion of the population classified into a clinically distinct risk group (i.e., a risk group that is meaningfully high or low risk from a clinical perspective). In the original model, less than 20 % of the population was classified into a clinically distinct risk group (19.2 %); this is the baseline proportion for the models with simulated predictors. Figure 2a, (left) shows the risk stratification for all models augmented with one simulated predictor. With one rare (5.0–10.0 % prevalence) simulated predictor added, none of the models classified 50 % of the population into clinically distinct risk groups, even with odds ratios of 16. With a more common predictor (20.0 % prevalence), an odds ratio of 8 was necessary to classify 50 % of the population into clinically distinct risk groups. One predictor of 40.0 % prevalence with an odds ratio of 6 was needed to classify 50 % of the population into a clinically distinct risk group, while an odds ratio of greater than 12 was needed to classify 75 % of the population into a clinically distinct risk group.

For models with three simulated predictors, shown in Fig. 2b, rare predictors (5.0–10.0 % prevalence) required odds ratios of 6 to 10, those with 20 % prevalence required odds ratios greater than 4, and common predictors (40.0 % prevalence) required odds ratios greater than 3 to classify 50 % of the population into clinically distinct risk groups. Models with rare predictors (5.0–10.0 % prevalence) were never able to classify 75 % of the cohort into clinically distinct risk group, though more common predictors did reach 75 % with odds ratios from 8 to 12.

Not surprisingly, models with five simulated predictors showed the best risk stratification performance, with lower required odds ratio and prevalence values (Fig. 2c). Five predictors of 5.0 % prevalence require odds ratios of 6 to classify 50 % of the cohort into clinically distinct strata and odds ratios of 12 to classify 75 % of the cohort. Models with 5 common predictors (20.0–40.0 % prevalence) classified 50 % of the population into clinically distinct risk groups with odds ratios of 4, and classified 75 % of the population with odds ratios of 8.

**Fig. 3:**
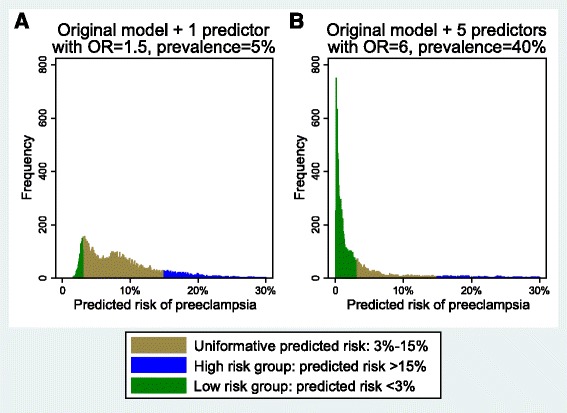
Histogram of predicted risk for each observation based on the original risk prediction model plus **a** one simulated predictor with an odds ratio of 1.5 and 5 % prevalence, and **b** five simulated predictors with odds ratios of 6 and 40 % prevalence. *Green bars* indicate a clinically distinct low risk group (predicted risk <3.0 %); *brown bars* indicate uninformative predicted risk (3.0–15.0 %); *blue bars* indicate a clinically distinct high risk group (predicted risk >15.0 %)

Figure 3 provides an alternative approach to examine the proportion of the population classified into clinically distinct risk groups. These histograms display the frequencies of different predicted risks according to two models with very different risk stratification capacities. In both sub-figures, the area in green (left) indicates the number of women with a predicted risk below 3.0 % (clinically distinct low risk group); the brown area (center) indicates the number of women with an uninformative predicted risk, not markedly different from the population average, or what we would predict for individuals based on a null model (3.0–15.0 %); the blue area (right) shows the number of women with predicted risk above 15.0 % (clinically distinct high risk group). The histogram on the left (Fig. 3a) shows predicted risks from a model with one simulated predictor added to the real data with an odds ratio of 1.5 and 5.0 % prevalence. The histogram on the right (Fig. 3b) shows predicted risks from a model with 5 simulated predictors with odds ratios of 6 and 40.0 % prevalence. A perfect model would classify 8.4 % of the population (the incidence of preeclampsia in this population) as high risk and the rest as low risk. As expected, the model on the left shows poor performance, with the majority of the population in the uninformative group (79.8 %), and far too few in the low risk group (8.6 %). The model on the right performs far better, and classifies the majority of the population (74.0 %) into a clinically distinct risk group. Appropriately, most (60.2 %) were classified into the low risk group, a small number were classified into the high risk group (13.8 %), and about a quarter (26.0 %) into the uninformative group.

**Fig. 4:**
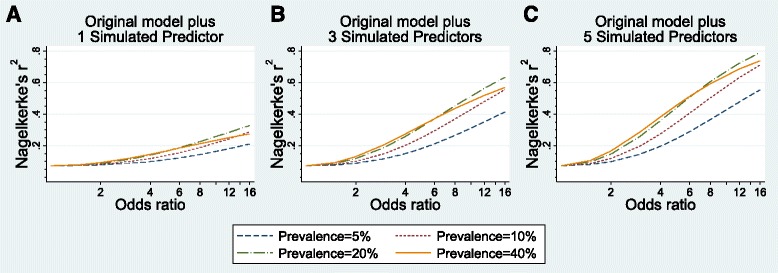
Overall model performance (measured by the proportion of variability in the outcome explained by the predictors, or Nagelkerge’s r^2^) of risk prediction models according to simulated predictor characteristics. The original risk prediction model was augmented with simulated predictors with prevalence from 5 to 40 % and odds ratios ranging from 1 to 16: **a** one added simulated predictor per model; **b** three added simulated predictors per model; **c** five added simulated predictors per model

The proportion of variability in preeclampsia risk that was explained by the predictors included in each model (Nagelkerke’s r^2^) was plotted according to predictor characteristics in Fig. 4. The observed predictors included in the original model explained very little of the variability in preeclampsia risk (7.2 %). As shown in Fig. 4a, models including only 1 simulated predictor showed poor performance, even when the added predictor was strongly associated with the outcome (OR = 16) and prevalent in the population (≥20.0 % prevalence). Model performance improved greatly with 3 and 5 added predictors. When 3 predictors with ≥20 % prevalence were included, models explained 50 % or more of the variability in preeclampsia risk when odds ratios were equal to 8 or more. With 5 added predictors, even uncommon predictors with large odds ratios (≥10) were able to explain more than 50 % of the outcome variability. As expected, models with 5 common (≥20.0 % prevalence) and odds ratios ≥12 demonstrated excellent performance, with r^2^ values approaching 75 %.

Table 3 combines the model performance measures of discrimination and risk stratification by presenting the proportion of the population classified into a stratum with an informative likelihood ratio. As the number, prevalence, and odds ratios of simulated predictors increase, model performance improves in terms of both discrimination and clinically relevant risk stratification capacity. This table illustrates a consistent relationship between discrimination according to the AUC and risk stratification capacity according to the proportion of the population classified into a clinically distinct risk group. Models that displayed minimum acceptable discriminative ability, assessed by an AUC of 0.7, exhibited poor risk stratification capacity, with 75 % of the population classified into a group that was clinically equivalent to the population baseline risk. In order to classify 50 % of the population into a clinically distinct risk group (high or low risk), AUCs of 0.85 were needed, while AUCs of 0.95 were needed for 75 % of the population to be classified into clinically distinct risk groups. A complete table with performance measures for all 120 models we built can be found in Additional file 2. These findings remained stable in our sensitivity analyses in we considered small changes in the thresholds used to define clinically distinct low and high risk groups.

**Table 3:** Model performance measures according to odds ratio, number, and prevalence of simulated predictors

Simulated predictor characteristics			Model performance measures			
OR of simulated predictors	Number of simulated predictors added to original model	Prevalence of simulated predictors	Proportion of population (%) with informative likelihood ratio ^a^	Proportion of population (%) assigned to clinically distinct risk group ^b^	AUC	Nagelkerke’s r^2^ (%)
2	3	10 %	0.0	27.2	0.71	10.0
2	3	20 %	0.0	29.9	0.73	11.7
2	3	40 %	0.0	34.8	0.74	12.9
2	5	10 %	0.0	28.9	0.73	11.8
2	5	20 %	0.0	33.3	0.75	14.6
2	5	40 %	0.0	39.2	0.77	16.6
6	3	10 %	0.0	54.4	0.83	29.4
6	3	20 %	63.6	63.6	0.87	37.1
6	3	40 %	70.2	70.0	0.88	37.1
6	5	10 %	66.9	66.9	0.88	40.6
6	5	20 %	72.0	72.0	0.92	50.7
6	5	40 %	73.8	73.8	0.93	51.2

^a^Defined as the proportion of the population classified into a stratum with a likelihood ratio <0.10 or >10.0

^b^Defined as the proportion of the population classified into a stratum with predicted risk meaningfully different than the baseline rate of pre-eclampsia in the population (<0.03 or >0.15)

## Discussion

Using the example of preeclampsia, our study established the predictor characteristics required for a risk prediction model to adequately discriminate cases from non-cases and to adequately classify patients into risk groups for whom distinct clinical management is warranted. Our approach of defining risk strata using clinically meaningful risk thresholds (rather than the more common method of using deciles of predicted risk) helped to establish the extent to which the application of the prediction models in clinical practice would likely influence clinical management decisions through improved identification of high and low risk patients. This approach helped to highlight that evaluation of a risk prediction model based on standard discrimination criteria alone may not provide a complete picture of the model’s clinical utility. We found that, if an AUC threshold of 0.7 were used to indicate acceptable risk prediction model performance, a substantial proportion of risk prediction models would be of limited use in clinical practice due to their poor risk stratification performance.

While our original data were population-based, and did not include novel clinical predictors, the characteristics of the preeclampsia predictors in our data are similar to those of other data sets with which researchers often aim to build risk prediction models. For example, a recently published prediction model for preeclampsia from a detailed clinical cohort included predictors with univariable odds ratios ranging from 0.5 to 2.9 (compared to 0.8 to 2.9 in our data) and prevalence values ranging from 3.9 to 50.3 % (compared to 0.4 to 43.3 %) [4]. Accordingly, the performance of our original model is expected to be similar to other models that aim to predict preeclampsia risk, and the findings from our simulation study are expected to be directly applicable to future work in this area.

Although risk stratification capacity is rarely the focus of risk prediction model performance evaluations, risk stratification capacity is central to the overall aim of risk prediction models [18]. Risk stratification involves transformation of continuous values of predicted risk into binary or categorical groups in which different levels of intervention or monitoring are warranted. We evaluated risk stratification capacity based on meaningful thresholds for identifying high and low risk patients (rather than arbitrary quantiles of risk in our study population) to maximize the clinical applicability of our findings. The primary method by which a clinical prediction model can improve health outcomes is by correctly classifying patients into groups with distinct clinical management plans (binary or categorical groups). For example, risk prediction models have been used to differentiate prostate cancer patients who would benefit from radical prostatectomy from those who need only receive annual screening tests [21], to differentiate children admitted to hospital with cerebrospinal fluid pleocytosis who would benefit from parenteral antibiotics from those who would not [22], and to identify children at high risk of abuse or neglect who would benefit from an early intervention strategy [23]. Thus, for a prediction model to change the course of a patient’s care, the model must perform well in terms of risk stratification capacity. We used a measure of risk stratification capacity that equally weighted a models’ ability to classify patients into low or high risk groups in order for this methodological analysis to be most broadly applicable. However, it is important to note that the clinical implications of misclassification of high risk patients into a low risk group are often not equal to the clinical implications of misclassification of low risk patients into a high risk group, and the relative importance of each depends on the specific research question.

Interestingly, the relationship between discrimination (AUC) and risk stratification capacity (proportion of the population classified into a clinically distinct risk group) was robust across our 120 models. Shown in Additional file 2, we see that a 0.7 AUC threshold for adequate discrimination is consistent with 20–30 % of the population being classified into clinically distinct risk groups. This held true even in our sensitivity analyses with slightly different definitions for clinically distinct risk groups. This calls into question the validity of an AUC of 0.7 indicating acceptable performance of a clinical prediction model. It is only when AUC values reach 0.8 or higher that any women were classified into a stratum with an informative likelihood ratio, and again, it is only when AUC values reach 0.85 that a sizeable proportion of the population (>40 %) is classified to a stratum with an informative likelihood ratio. While 0.7 is widely accepted as the lower limit of acceptable discrimination, this threshold originated from a footnote of an early study of prediction model performance [15], and has not since been formally evaluated. Our findings suggest that a higher AUC threshold (above 0.8) better indicate a model’s clinical utility, although further research is needed to formally identify the most appropriate threshold value.

The calibration of our original model was adequate, as measured by the Hosmer-Lemeshow goodness-of-fit test and visual comparison of observed versus predicted risks. Our findings are applicable to models with adequate calibration. Models with poor calibration should not be used for risk prediction [20].

The findings of this simulation study must be interpreted in light of several limitations. First, evaluation of a prediction model’s risk stratification capacity is heavily dependent on the clinical context for the particular research question at hand. For example, if classification into a high risk group would lead a clinician to perform benign intervention, the threshold for defining the risk group would be less stringent than for an intervention that carries potential harms or side effects. Thus, the particular thresholds we used to define clinically distinct risk groups for this simulation study were based on the clinical context of preeclampsia diagnosis and management, and may not be generalizable to other research questions. However, we do expect the broad take-home message of our findings to be generalizable to a wider array of clinical contexts, including prediction of outcomes that are not as rare as preeclampsia.

To preserve interpretability of our results, we did not build risk prediction models to simulate all situations that a research team may encounter. The predictors we simulated were binary and independent of one another, and all regression models were logistic. Continuous predictors may, in some cases, result in better predictive ability than binary predictors. Conversely, non-independent predictors may need to be more prevalent in the population and/or have higher univariable odds ratios with the outcome to achieve the same model performance we report. While risk prediction models are most often based on logistic regression models, extensions of this work to other model types, such as linear regression models or Cox proportional hazards models, merit further investigation.

## Conclusions

Our findings can serve as a guide to researchers who seek to develop a risk prediction model. In particular, by examining the relationship between predictors’ univariable odds ratios and prevalences and model performance, researchers and peer reviewers should be able to estimate a range of expected model performance parameters before a model has been built. This form of guidance has not yet been available to researchers, and may lead to increased efficiency of research efforts and funds.

## Additional files

Additional file 1: Presents the observed and optimism-correct area under the receiver-operator characteristic curve for risk prediction models built for 12 pregnancy and birth outcomes, and provides a complete list of included predictors for each model. (DOCX 30 kb) Additional file 2: Presents all model performance measures for each of the 120 models built using data augmented with simulated predictors. Performance measures include the area under the receiver-operator characteristic curve, the proportion of the population classified into a clinically distinct risk group, the proportion of the population with an informative likelihood ratio, and Nagelkerke’s r^2^. (DOCX 47 kb)
