# Supplementary material for: Predictor characteristics necessary for building a clinically useful risk prediction model: a simulation study
Source: BMC Med Res Methodol. 2016 Sep 21;16:123. doi: 10.1186/s12874-016-0223-2 (PMC5031287; doi:10.1186/s12874-016-0223-2)
Supplement: Additional file 1: — Presents the observed and optimism-correct area under the receiver-operator characteristic curve for risk prediction models built for 12 pregnancy and birth outcomes, and provides a complete list of included predictors for each model. (DOCX 30 kb) [file 12874_2016_223_MOESM1_ESM.docx]

| **Additional File 1. Performance of prediction models: Area under the receiver-operator characteristic curve, apparent and corrected for optimism.** | | |
| --- | --- | --- |
|  | Apparent area under ROC curve | Optimism-corrected area under ROC curve |
| Preeclampsia | 0.68 | 0.68 |
| Gestational diabetes | 0.66 | 0.66 |
| Macrosomia | 0.64 | 0.62 |
| Shoulder dystocia | 0.60 | 0.59 |
| Spontaneous preterm delivery <32 weeks | 0.59 | 0.57 |
| Indicated preterm delivery <37 weeks | 0.62 | 0.62 |
| Cesarean delivery | 0.61 | 0.60 |
| Postpartum hemorrhage requiring intervention to control bleeding | 0.59 | 0.58 |
| Maternal mortality or severe morbidity | 0.59 | 0.58 |
| Stillbirth | 0.60 | 0.58 |
| NICU stay ≥48 hours | 0.61 | 0.60 |
| In-hospital newborn mortality | 0.61 | 0.60 |
| All models included prepregnancy body mass index, maternal age, height, parity, and smoking. Models for preeclampsia did not include preexisting hypertension; models for gestational diabetes did not include pre-gestational diabetes (type 1 or 2). Models for cesarean delivery were restricted to nulliparous women.  Outcome-specific predictors were as follows:  Macrosomia and shoulder dystocia: prior macrosomic baby, prior stillbirth, prior neonatal death, number of spontaneous abortions  Spontaneous and indicated preterm delivery, stillbirth, NICU stay ≥48 hours; in-hospital newborn death: prior stillbirth, prior neonatal death, number of spontaneous abortions | | |
