# Supplementary material for: Predictor characteristics necessary for building a clinically useful risk prediction model: a simulation study
Source: BMC Med Res Methodol. 2016 Sep 21;16:123. doi: 10.1186/s12874-016-0223-2 (PMC5031287; doi:10.1186/s12874-016-0223-2)
Supplement: Additional file 2: — Presents all model performance measures for each of the 120 models built using data augmented with simulated predictors. Performance measures include the area under the receiver-operator characteristic curve, the proportion of the population classified into a clinically distinct risk group, the proportion of the population with an informative likelihood ratio, and Nagelkerke’s r2. (DOCX 47 kb) [file 12874_2016_223_MOESM2_ESM.docx]

| **Additional File 2. Performance measures of risk prediction models for preeclampsia according to odds ratio, number, and prevalence of simulated predictors** | | | | | |  |
| --- | --- | --- | --- | --- | --- | --- |
| **Simulated predictor characteristics** | | | **Model performance measures** | | |  |
| OR of simulated predictors | Number of simulated predictors added to original model | Prevalence of simulated predictors (%) | Area under the receiver-operator characteristic curve | Proportion of population classified into a clinically distinct risk group (%) | Proportion of population with informative likelihood ratio** | Nagelkerke’s r^2^  (%) |
| 1.5 | 1 | 5% | 0.68 | 20.0 | 0.0 | 7.4 |
| 1.5 | 1 | 10% | 0.68 | 20.5 | 0.0 | 7.5 |
| 1.5 | 1 | 20% | 0.69 | 21.4 | 0.0 | 7.7 |
| 1.5 | 1 | 40% | 0.69 | 22.0 | 0.0 | 7.9 |
| 1.5 | 3 | 5% | 0.69 | 21.1 | 0.0 | 7.7 |
| 1.5 | 3 | 10% | 0.69 | 22.4 | 0.0 | 8.1 |
| 1.5 | 3 | 20% | 0.70 | 24.0 | 0.0 | 8.7 |
| 1.5 | 3 | 40% | 0.70 | 26.0 | 0.0 | 9.2 |
| 1.5 | 5 | 5% | 0.69 | 22.1 | 0.0 | 8.0 |
| 1.5 | 5 | 10% | 0.70 | 23.5 | 0.0 | 8.7 |
| 1.5 | 5 | 20% | 0.71 | 25.8 | 0.0 | 9.7 |
| 1.5 | 5 | 40% | 0.71 | 28.2 | 0.0 | 10.5 |
| 2 | 1 | 5% | 0.69 | 21.0 | 0.0 | 7.8 |
| 2 | 1 | 10% | 0.69 | 22.6 | 0.0 | 8.2 |
| 2 | 1 | 20% | 0.70 | 25.1 | 0.0 | 8.8 |
| 2 | 1 | 40% | 0.70 | 26.6 | 0.0 | 9.2 |
| 2 | 3 | 5% | 0.70 | 22.9 | 0.0 | 8.8 |
| 2 | 3 | 10% | 0.71 | 27.2 | 0.0 | 10.0 |
| 2 | 3 | 20% | 0.73 | 30.0 | 0.0 | 11.7 |
| 2 | 3 | 40% | 0.74 | 34.8 | 0.0 | 12.9 |
| 2 | 5 | 5% | 0.71 | 26.2 | 0.0 | 9.7 |
| 2 | 5 | 10% | 0.73 | 28.9 | 0.0 | 11.8 |
| 2 | 5 | 20% | 0.75 | 33.3 | 0.0 | 14.6 |
| 2 | 5 | 40% | 0.77 | 39.1 | 0.0 | 16.6 |
| 3 | 1 | 5% | 0.69 | 22.9 | 0.0 | 8.7 |
| 3 | 1 | 10% | 0.71 | 26.3 | 0.0 | 9.9 |
| 3 | 1 | 20% | 0.72 | 31.9 | 0.0 | 11.3 |
| 3 | 1 | 40% | 0.73 | 35.3 | 0.0 | 12.0 |
| 3 | 3 | 5% | 0.72 | 29.6 | 0.0 | 11.7 |
| 3 | 3 | 10% | 0.75 | 35.4 | 0.0 | 15.0 |
| 3 | 3 | 20% | 0.78 | 39.3 | 0.0 | 18.8 |
| 3 | 3 | 40% | 0.80 | 49.8 | 0.0 | 20.7 |
| 3 | 5 | 5% | 0.75 | 34.5 | 0.0 | 14.5 |
| 3 | 5 | 10% | 0.79 | 38.2 | 0.0 | 19.7 |
| 3 | 5 | 20% | 0.83 | 49.3 | 0.0 | 25.8 |
| 3 | 5 | 40% | 0.85 | 51.2 | 37.5 | 28.6 |
| 4 | 1 | 5% | 0.70 | 24.0 | 0.0 | 9.9 |
| 4 | 1 | 10% | 0.72 | 28.9 | 0.0 | 11.8 |
| 4 | 1 | 20% | 0.74 | 36.8 | 0.0 | 14.0 |
| 4 | 1 | 40% | 0.75 | 42.1 | 0.0 | 14.5 |
| 4 | 3 | 5% | 0.74 | 33.6 | 0.0 | 14.9 |
| 4 | 3 | 10% | 0.78 | 42.3 | 0.0 | 20.1 |
| 4 | 3 | 20% | 0.82 | 50.1 | 0.0 | 25.9 |
| 4 | 3 | 40% | 0.83 | 58.3 | 20.1 | 27.4 |
| 4 | 5 | 5% | 0.78 | 41.2 | 0.0 | 19.6 |
| 4 | 5 | 10% | 0.83 | 49.2 | 0.0 | 27.5 |
| 4 | 5 | 20% | 0.87 | 59.7 | 59.7 | 35.9 |
| 4 | 5 | 40% | 0.89 | 62.1 | 62.1 | 38.2 |
| 5 | 1 | 5% | 0.71 | 25.2 | 0.0 | 11.1 |
| 5 | 1 | 10% | 0.73 | 31.2 | 0.0 | 13.7 |
| 5 | 1 | 20% | 0.76 | 40.5 | 0.0 | 16.4 |
| 5 | 1 | 40% | 0.77 | 48.2 | 0.0 | 16.6 |
| 5 | 3 | 5% | 0.76 | 36.9 | 0.0 | 18.1 |
| 5 | 3 | 10% | 0.81 | 48.2 | 0.0 | 25.0 |
| 5 | 3 | 20% | 0.85 | 58.3 | 3.6 | 31.9 |
| 5 | 3 | 40% | 0.86 | 64.5 | 47.6 | 32.7 |
| 5 | 5 | 5% | 0.81 | 46.9 | 0.0 | 24.5 |
| 5 | 5 | 10% | 0.86 | 60.0 | 33.4 | 34.5 |
| 5 | 5 | 20% | 0.90 | 69.0 | 69.0 | 44.1 |
| 5 | 5 | 40% | 0.91 | 68.3 | 68.3 | 45.6 |
| 6 | 1 | 5% | 0.72 | 26.2 | 0.0 | 12.2 |
| 6 | 1 | 10% | 0.75 | 33.3 | 0.0 | 15.5 |
| 6 | 1 | 20% | 0.78 | 44.0 | 0.0 | 18.7 |
| 6 | 1 | 40% | 0.78 | 54.5 | 0.0 | 18.4 |
| 6 | 3 | 5% | 0.78 | 40.0 | 0.0 | 21.1 |
| 6 | 3 | 10% | 0.83 | 54.4 | 0.0 | 29.4 |
| 6 | 3 | 20% | 0.87 | 63.6 | 63.6 | 37.1 |
| 6 | 3 | 40% | 0.88 | 70.0 | 70.0 | 37.1 |
| 6 | 5 | 5% | 0.83 | 52.6 | 0.0 | 28.9 |
| 6 | 5 | 10% | 0.88 | 66.9 | 66.9 | 40.6 |
| 6 | 5 | 20% | 0.92 | 72.0 | 72.0 | 50.7 |
| 6 | 5 | 40% | 0.93 | 73.8 | 73.8 | 51.2 |
| 8 | 1 | 5% | 0.73 | 27.9 | 0.0 | 14.3 |
| 8 | 1 | 10% | 0.77 | 36.6 | 0.0 | 18.8 |
| 8 | 1 | 20% | 0.80 | 49.6 | 0.0 | 22.6 |
| 8 | 1 | 40% | 0.80 | 64.1 | 0.0 | 21.3 |
| 8 | 3 | 5% | 0.81 | 45.1 | 0.0 | 26.5 |
| 8 | 3 | 10% | 0.87 | 67.2 | 57.5 | 36.9 |
| 8 | 3 | 20% | 0.90 | 70.7 | 70.7 | 45.3 |
| 8 | 3 | 40% | 0.90 | 76.6 | 76.6 | 43.6 |
| 8 | 5 | 5% | 0.86 | 65.1 | 16.0 | 36.6 |
| 8 | 5 | 10% | 0.91 | 74.7 | 74.7 | 50.3 |
| 8 | 5 | 20% | 0.94 | 80.6 | 80.6 | 60.6 |
| 8 | 5 | 40% | 0.94 | 81.3 | 81.3 | 59.3 |
| 12 | 1 | 5% | 0.75 | 30.0 | 0.0 | 18.0 |
| 12 | 1 | 10% | 0.79 | 41.7 | 0.0 | 24.3 |
| 12 | 1 | 20% | 0.83 | 60.6 | 0.0 | 28.5 |
| 12 | 1 | 40% | 0.83 | 74.1 | 51.4 | 25.1 |
| 12 | 3 | 5% | 0.84 | 54.0 | 12.6 | 35.0 |
| 12 | 3 | 10% | 0.90 | 80.9 | 80.9 | 47.9 |
| 12 | 3 | 20% | 0.93 | 76.4 | 76.4 | 56.3 |
| 12 | 3 | 40% | 0.93 | 81.9 | 81.9 | 51.8 |
| 12 | 5 | 5% | 0.90 | 81.7 | 81.7 | 47.8 |
| 12 | 5 | 10% | 0.95 | 79.7 | 79.7 | 63.2 |
| 12 | 5 | 20% | 0.96 | 88.5 | 88.5 | 72.4 |
| 12 | 5 | 40% | 0.96 | 88.2 | 88.2 | 68.6 |
| 16 | 1 | 5% | 0.76 | 31.7 | 0.0 | 21.1 |
| 16 | 1 | 10% | 0.81 | 45.4 | 0.0 | 28.7 |
| 16 | 1 | 20% | 0.85 | 71.1 | 22.0 | 32.7 |
| 16 | 1 | 40% | 0.84 | 78.8 | 55.3 | 27.5 |
| 16 | 3 | 5% | 0.89 | 63.0 | 12.6 | 41.3 |
| 16 | 3 | 10% | 0.92 | 87.2 | 87.2 | 55.4 |
| 16 | 3 | 20% | 0.95 | 79.8 | 79.8 | 63.3 |
| 16 | 3 | 40% | 0.94 | 84.1 | 84.4 | 56.8 |
| 16 | 5 | 5% | 0.92 | 88.7 | 88.7 | 55.4 |
| 16 | 5 | 10% | 0.96 | 83.0 | 83.0 | 71.2 |
| 16 | 5 | 20% | 0.97 | 91.6 | 91.6 | 79.0 |
| 16 | 5 | 40% | 0.97 | 91.0 | 91.0 | 73.8 |
| *Proportion of the population with informative predicted risk is defined as the proportion of the population classified into a stratum with predicted risk <0.03 or >0.15  ** Proportion of the population with informative likelihood ratio is defined as the proportion of the population classified into a stratum with a likelihood ratio <0.10 or >10.0. | | | | | | |
